# Of funding and finches

**DOI:** 10.1186/s13059-019-1796-y

**Published:** 2019-08-28

**Authors:** Verena Jantsch, Michael Jantsch

**Affiliations:** 1 Department of Chromosome Biology, Max Perutz Labs, University of Vienna, Vienna BioCenter, Dr. Bohrgasse 9, A- 1030 Vienna, Austria; 2 Division of Cell- and Developmental Biology, Center of Anatomy and Cell Biology, Medical University of Vienna, Schwarzspanierstrasse 17, A-1090 Vienna, Austria

## Abstract

Funding research is a challenge faced by most scientists around the world. Genome Biology has invited four scientists based in three different countries to share their own experience and opinions regarding funding, the difficulties young scientists must overcome, and how the process of securing funding can be improved. In this part, Verena and Michael Jantsch describe their thoughts on funding research from a European perspective.

## Main text

Verena Jantsch is a faculty member at the Max Perutz Labs (University of Vienna), where she studies meiosis in the *Caenorhabditis elegans* model. She currently holds a Professorship of Eukaryotic Genetics. Michael Jantsch is Professor for Cell and Developmental Biology at the Medical University of Vienna.

In the life sciences, the number of institutional, private and public funding bodies vary largely across different countries. Similarly, the overall volume of funding available and the ties attached to eligibility for particular funding schemes vary greatly. For instance, governmental research and development (R&D) expenses are the highest in South Korea, which spends a little over 4.55% of its gross domestic product (GDP) on R&D, followed by Switzerland (3.38%) and Japan (3.2%). Surprisingly, some scientifically highly renowned countries spend much less; the UK spends only 1.66% of its GDP on R&D [1]. It should be remembered that these numbers need to be taken lightly, as the number and volume of private funding bodies and the definition of what qualifies as R&D spending differ across countries.

However, for a successful career in science, an appropriate research surrounding and reachable funding opportunities are vital when making the first steps as a young group leader and for becoming established in the scientific community. Consequently, countries with solid and transparent research funding, a public appreciation for science, and transparent career models are the first choice for young scientists. Interestingly, however, the strategies of how to become competitive for a young investigator’s position within Europe has changed in recent years, not least due to a changing funding environment. These changes also have implications on strategic decisions during undergraduate and graduate studies.

Students’ access to European universities is largely handled at the national level with different barriers in different countries, including the different languages in which undergraduate students are taught. Moreover, types of studies and length of education can vary considerably, resulting in heterogeneous experiences and scientific output at the graduate level. To compensate for the different qualities of training, it was common to take one or even two extensive postdoctoral positions in prestigious research institutes that frequently lasted several years. Such extensive postdoctoral stays could help to achieve visibility through multiple publications and establish a broad research portfolio that would be attractive for hiring committees at high-quality research institutes and the corresponding national funding sources alike.

However, in recent years, by establishing pan-European science funding bodies, the European Union has tried to level out national differences in funding schemes within Europe. Here, the European Research Council (ERC) grants are especially attractive to support individual research projects at all stages of a scientific career [2]. In fact, obtaining any of the three ERC research grants (starting, consolidator, or advanced) has become a major factor in building or advancing a career in science, as these prestigious grants not only provide good support for competitive research but have become an asset to research institutions, thereby also affecting the hiring policy of these institutions.

Interestingly, this change in hiring policy also affects the training and education paths of young scientists. To be eligible for an ERC starting grant, the candidate needs to be no more than 7 years beyond obtaining their PhD. It is therefore increasingly important to pull together numerous high-impact publications within a limited number of years. With this in mind, it has become more advisable to extend PhD studies to obtain several publications before obtaining the PhD degree, followed by a single postdoctoral position in order to still be eligible for several rounds of application for an ERC starting grant. This also increases the desirability to perform one’s undergraduate and graduate studies at places that increase the likelihood of achieving respectable publications. It is obvious that national differences in the education system, including universities, can be a limiting factor in this game. Moreover, extended PhD studies sometimes contrast with national PhD programmes that aim for short and concise training programmes, whereby it is expected that “elite students” finish their studies, including publication of one, if not more, first author papers within 3 to 4 years. The often lengthy time from first submission of a manuscript until its acceptance shows the difficulties of this “arms race” and highlights the importance of making the right strategic choice when applying for a PhD position, as this may have long-lasting effects for one’s future career. It remains to be shown how much the eligibility for an ERC junior grant and its impact on hiring policies for junior faculty positions will affect scientific diversity and “out of the box” careers. It may show that unconventional careers and unconventional ideas are becoming less popular when competing for elite grants.

Also, further up the faculty level, the acquisition of an ERC advanced grant has become a career-shaping tool. This might be for competitive negotiations (within or outside a scientist’s research institution), for climbing up the career ladder, for more laboratory space, or more technical and financial support. Today, many universities will consider an ERC grant holder a prestigious addition to their faculty. While scientific quality is with no doubt a key factor for obtaining an ERC consolidator grant, scientific age will again be a major factor. ERC advanced grant boards judge one’s standing in the field but also a candidate’s involvement in “science policy”, which goes hand in hand with networking skills. Achievements, such as conference organization, editorial activity, or duties as dean or chairperson, are considered factors that represent the applicant’s scientific merit. It needs to be recognized that such “add-on” requirements strongly rely on the capacity of a researcher to dedicate time towards these duties. This may contrast to the time dedicated to developing teaching concepts, to mentor students, or even to raising children. The current criteria used to consider a proposal may put individuals with limited capacities for networking and organizational activities into a less competitive position irrespective of their scientific originality.

When Charles Darwin reached the Galapagos Islands during the second voyage of the Beagle, he collected birds whose beak forms varied widely [3]. What later became known as Darwin’s finches, and one of the most popularized examples for speciation, turned out to be the outcome of selective pressure to grow on specialized food sources [4]. As funding is metaphorically the major food source for scientist’s careers, funding agencies need to be aware of their responsibilities and should consider that guidelines will transform researchers and their careers as much as research institutions and research itself. Care should be taken that the quest for funding does not only foster “fulfillers of requirements” but indeed gives the freedom and time required to develop novel and innovative research ideas. As scientists, we all like to cite serendipitous discoveries to justify basic research. We should all live up to these standards in granting and hiring committees and should be aware that the outcome of good science cannot be predicted by metric standards.
